# Ethical Knowledge, Challenges, and Institutional Strategies Among Medical AI Developers and Researchers: Focus Group Study

**DOI:** 10.2196/79613

**Published:** 2026-01-28

**Authors:** Sophia Fantus, Jinxu Li, Tianci Wang, Lu Tang

**Affiliations:** 1 School of Social Work The University of Texas at Arlington Arlington, TX United States; 2 Department of Communication & Journalism Texas A&M University College Station, TX United States; 3 Burnett School of Medicine Texas Christian University Fort Worth, TX United States

**Keywords:** AI ethics, AI, artificial intelligence, ethics, focus group, medical

## Abstract

**Background:**

As artificial intelligence (AI) becomes increasingly embedded in clinical decision-making and preventive care, it is urgent to address ethical concerns such as bias, privacy, and transparency to protect clinician and patient populations. Although prior research has examined the perspectives of medical AI stakeholders, including clinicians, patients, and health system leaders, far less is known about how medical AI developers and researchers understand and engage with ethical challenges as they develop AI tools. This gap is consequential because developers’ ethical awareness, decision-making, and institutional environments influence how AI tools are conceptualized and deployed in practice. Thus, it is essential to understand how developers perceive these issues and what supports they identify as necessary for ethical AI development.

**Objective:**

The objectives of the study were twofold: (1) to examine medical AI developers’ and researchers’ knowledge, attitudes, and experiences with AI ethics; and (2) to identify recommendations to enhance and strengthen interpersonal and institutional ethics-focused training and support.

**Methods:**

We conducted 2 semistructured focus groups (60-90 minutes each) in 2024 with 13 AI developers and researchers affiliated with 5 US-based academic institutions. Participants’ work spanned a wide variety of medical AI applications, including Alzheimer disease prediction, clinical imaging, electronic health records analysis, digital health, counseling and behavioral health, and genotype–phenotype modeling. Focus groups were conducted via Microsoft Teams, recorded, and transcribed verbatim. We applied conventional qualitative content analysis to inductively identify emerging concepts, categories, and themes. Coding was performed independently by 3 researchers, with consensus reached through iterative team meetings.

**Results:**

The analysis identified four key themes: (1) AI ethics knowledge acquisition: participants reported learning about ethics informally through peer-reviewed literature, reviewer feedback, social media, and mentorship rather than through structured training; (2) ethical encounters: participants described recurring ethical challenges related to data bias, patient privacy, generative AI use, commercialization pressures, and a tendency for research environments to prioritize model accuracy over ethical reflection; (3) reflections on ethical implications: participants expressed concern about downstream effects on patient care and clinician autonomy, and model generalizability, noting that rapid technological innovation outpaces regulatory and evaluative processes; and (4) strategies to mitigate ethical concerns: recommendations included clearer institutional guidelines, ethics checklists, interdisciplinary collaboration, multi-institutional data sharing, enhanced institutional review board support, and the inclusion of bioethicists as members of the AI research team.

**Conclusions:**

Medical AI developers and researchers recognize significant ethical challenges in their work but lack structured training, resources, and institutional mechanisms to address them. Findings of this study underscore the need for institutions to consider embedding ethics into research processes through practical tools, mentorship, and interdisciplinary partnerships. Strengthening these supports is essential to preparing the next generation of developers to design and deploy ethical AI in health care.

## Introduction

### Artificial Intelligence in Health and Medicine

Artificial intelligence (AI) is reshaping health care; AI tools are aimed at reducing costs [1], streamlining clinical workflow [2], and facilitating clinician and patient experiences [3]. Current AI applications may include assistance with clinical decisions, image-based diagnosis, self-diagnosis, mental health screening, and chronic disease management [2]. For example, electronic health records (EHRs) use natural language processing to support clinical decisions [4], and at-home AI monitoring systems assist older adults and those with long-term chronic illnesses, potentially alleviating caregiver burden [5]. Health care providers have started to use AI for medical imaging, diagnosis and disease screening, and prediction [6-9]. Furthermore, emerging scholarship demonstrates that AI has shown, to some extent, faster diagnostic speed and higher accuracy than human experts in image analysis and precision medicine [1,10]. The speed at which AI has been integrated and accepted into health care networks and its ease of access for users are unprecedented.

Despite these benefits, there are significant obstacles to AI implementation in clinical practice. Concerns about patient data security, privacy, clinician and patient autonomy, and decision-making may erode trust in AI outputs [11,12]. In addition, the pace of AI technology innovation often surpasses regulatory guidance at the federal and state levels [13]. Identifying and understanding ethical challenges may help establish practice and policy guidelines across health systems. Such mechanisms may ensure that AI developers and researchers, along with clinicians, patients, and health system leadership and administration, adapt and integrate AI that considers the needs and perspectives of all stakeholders. The purpose of this study was to explore AI developers’ and researchers’ understanding of and training in medical AI ethics.

### Ethical Attitudes and Knowledge of Medical AI

Recent scholarship has shown rising ethical concerns among various stakeholders such as clinicians, patients, families, and policymakers who engage with medical AI [12,14-16]. Clinicians report that AI tools serve as a time-saving benefit in completing administrative tasks, which may effectively increase clinical productivity and patient engagement [6,17]. Yet, they are concerned with patient data privacy, the impacts on the clinician-patient relationship, and the possibility that the financial burdens of AI tools may heighten health inequities [7,17,18]. Patients and families voice similar concerns, focusing on patient autonomy and shared decision-making [19,20]. Patients articulate unease with the application of AI in treatment recommendations, medication administration, and surgical procedures [20].

### Gap in Scholarship

The attitudes and perspectives of medical AI ethics among health care stakeholders are important. Yet, perspectives of other key stakeholders, such as AI developers and researchers, are underrepresented in research [12]. Previous studies demonstrate that ethical issues, including privacy and data security, fairness, transparency, and reliability of machine learning predictive analytics, are encountered by AI developers [21-23]. Yet, the perceived responsibility of developers and researchers to mitigate potential harms varies widely; some AI developers are cognizant of the broader societal impacts of AI (beyond technical considerations and optimization), while others feel disconnected and detached from direct patient and clinician outcomes [14,24,25]. Many AI developers report barriers to mitigating AI harms, including limited authority to make such decisions, and external pressures to deliver products quickly, all of which can hinder ethical reflexivity [26].

With a paucity of evidence-informed data on developers’ AI knowledge and attitudes, further research is necessary to understand how AI ethics is addressed prior to deployment. Academic institutions play a central role in AI research and development, which lays the foundation for industry’s AI design and application [14,24]. Academia has an important role in educating, training, and shaping the future generation of AI developers. This study presents a unique opportunity to guide policy, practice, and education efforts in research institutes that are aligned with the needs of AI developers and consider the deployment of ethical AI across health systems.

### Research Rationale

AI developers and researchers work on algorithms that ultimately shape medical AI tools. Yet, clinicians often assume that AI tools used in clinical settings have been ethically scrutinized prior to deployment [27]. To understand ethical encounters of AI design, this study identifies the knowledge, attitudes, and training in medical AI ethics among AI researchers and developers. As an exploratory, pilot study, this work aims to offer an initial, in-depth understanding of how developers and researchers experience and navigate ethical challenges in medical AI. Rather than seeking generalizability, our goal was to capture diverse perspectives across academic contexts to illuminate key issues and inform the design of future large-scale, quantitative investigations on AI ethics training and institutional practices. Findings may inform strategies to facilitate AI ethics integration in development.

## Methods

### Recruitment and Sampling

The research team qualitatively explored perspectives of medical AI ethics among AI researchers and developers who were employed at academic institutions in the United States. Members of the research team had expertise in health communications, AI and health promotion, and bioethics. Participants’ inclusion criteria were (1) aged ≥18 years; (2) ability to read, understand, and communicate in English; (3) employed at an academic institution; (4) involved in medical AI research and development; and (5) consent to participate in a focus group. Participants were recruited through purposive sampling and chain referral methods to appropriately reach individuals who had an academic background in working with medical AI tools and the creation of algorithms. A study announcement and blurb were sent through listserv emails, through networks and contacts of the research team, and by word-of-mouth. Participants responded by email, and a focus group was scheduled that met participants’ availability through an anonymous When2Meet Poll.

### Data Collection

Focus groups (n=2; 60 minutes each) were held over Microsoft Teams and facilitated by the lead author. The facilitator had no pre-existing relationships with any participants. The lead author introduced herself to the research team and explained her background in clinical ethics, bioethics, and health scholarship prior to initiation. A semistructured interview guide was used in each focus group to reflect on participants’ knowledge, attitudes, and encounters with AI ethics, and practical strategies to enhance or improve ethics education and training. Participants did not receive the interview guide prior to the scheduled focus group. Some example questions included the following: (1) What do you think is the extent of your AI ethics knowledge? (2) What is your prior experience with AI ethics? (3) What are the ethical concerns you have when conducting AI research? (4) How as a research team do you deliberate ongoing ethical issues you face? (5) In your current workplace, what training or learning opportunities are there with AI ethics? (6) What can your supervisor or the institution or university do to support you in understanding and identifying ethical issues in AI research? The interview guide was developed and piloted by members of the research team.

All focus groups (roughly 60-90 minutes each) were recorded and transcribed verbatim. No repeat interviews or focus groups were conducted. Transcripts were cleaned for errors and deidentified to protect participant confidentiality. Recordings and transcripts were stored on the university’s secure password-protected server, and only members of the research team had access to the data. Thematic saturation was assessed through iterative review during and after the second focus group. At that stage, no substantively new themes emerged, and only minor variations of existing concepts were observed. We therefore determined that thematic saturation had been sufficiently achieved for the purposes of this pilot, exploratory study, and data collection concluded accordingly. In addition, members of the research team contacted different principal investigators within their respective academic units to enhance diversity in disciplinary backgrounds, institutional affiliations, and research areas. This strategy broadened the participant pool and helped capture a wider range of perspectives on AI ethics while maintaining the feasibility of this exploratory qualitative study.

### Data Analysis

We applied conventional content analysis, an inductive qualitative approach used when limited theory or research exists on a topic [28]. Analysis occurs directly from the data without the use of preconceived frameworks or codebooks, allowing researchers to conduct in-depth exploration of raw data [28]. All focus group transcripts (roughly 70 pages of transcript data) were redacted and anonymized, and participants were given a specific numerical code to ensure the accuracy of responses. The 2 focus group transcripts were disseminated to 3 members of the research team (known as the coding team) to review independently from one another. Transcripts were reviewed, and data were coded inductively to form new insights and perspectives on medical AI ethics. Initial coding was conducted first to highlight exact words or phrases to denote emerging concepts. The whole research team met to discuss initial thoughts and impressions from the transcript and to develop a codebook. The transcripts were reviewed a few more times by the three independent coders to organize: (1) codes into categories, (2) categories into clusters, and (3) clusters into emerging themes. The coding team met frequently to finalize the codebook and to reach consensus on emerging themes and patterns from the data. Discrepancies in coding were discussed by the coding team to reach consensus. Codes were iteratively clustered into broader conceptual categories and then synthesized into higher-order themes that reflected shared meanings across participants. Throughout this process, the team also noted and discussed negative or divergent cases to ensure that contrasting perspectives were represented and that the final themes captured the full range of participant experiences. Once the codebook was finalized, the whole research team met to review findings and finalize themes. Rigor and trustworthiness were attained through peer debriefing with other AI and data science experts. The themes were grounded in participant data to capture their perspectives, thoughts, and insights on medical AI ethics.

### Ethical Considerations

The study was approved by the Institutional Review Boards (IRBs) of Texas A&M University (approval number: IRB2023-0396D) and the University of Texas at Arlington (approval number: 2023-0234) prior to participant recruitment. Before the scheduled focus groups, participants received a copy of an informed consent form, which they signed and returned electronically. At the beginning of each focus group, the research team reviewed the consent information again orally, provided time for clarifying questions, and reiterated confidentiality limits and group norms. To protect the privacy and confidentiality of the participants, we transcribed the video recordings and removed all identifying information, including names, geographic location, or university affiliations. We conducted the data analysis based on the anonymized transcripts instead of videos. Each participant was paid US $20 in an Amazon gift card as compensation for their time.

## Results

### Overview

We interviewed a total of 13 participants employed in medical AI research and development. No participants declined or withdrew participation either before or during the focus group. Six (46%) participants were women, and 7 (54%) were men. Six (46%) participants identified as Chinese, and other participants identified as Asian Indian, Middle Eastern, Egyptian, Bangladeshi, Pacific Islander, or Taiwanese; only 1 participant identified as White. Participants held a range of positions, including research faculty (5/13, 38%), graduate students or research assistants (7/13, 54%), and programmer (1/13, 0.07%). Focus group participants represented 5 distinct academic institutes from different regions in the United States. Participants’ AI research included (1) disease or surgery outcomes prediction, (2) prediction and optimization of treatment, (3) analysis of electronic medical records or diagnostic imaging, (4) genetic analysis and genotype–phenotype correlation, (5) AI in counseling and behavioral health, and (6) AI in digital health and clinical trial work.

Four themes emerged from the analysis of focus group transcripts: (1) AI ethics knowledge acquisition that demonstrates how and where participants obtain AI ethics information, (2) ethical encounters that identify the main ethical issues that arise from algorithm development and design, (3) perceptions of ethical encounters to understand the implications of unresolved ethical encounters, and (4) recommendations and strategies to facilitate ethical deliberation and debrief in the workplace (Table 1).

**Table 1:** Themes and examples identified through conventional content analysis of focus groups on medical artificial intelligence (AI) ethics among US-based AI developers and researchers (2024).

Themes (innovation-decision process) and subthemes		Description	Quote
**AI knowledge acquisition** **(knowledge)**			
	Peer-reviewed publications	Learning about AI ethics from published studies discussing bias, fairness, and responsible AI.	“So I was following up some publications, so I started to see the trend of new publications coming up and talking about like as I mentioned AI bias…”
	Reviewers’ feedback	Gaining awareness of ethical issues through reviewers’ comments during the publication process.	“The first time I realized it was when I submitted my manuscript to Nature-like journals; most reviewers pointed it out, and that’s when I started thinking about ethics seriously. ”
	Social media and AI policy updates	Following experts and organizations online to stay current with national and international AI ethics guidelines.	“I don’t know if you’ve seen the recent news, like DeepMind’s phone app for ChatGPT.”
	Informal mentorship and seminars	Receiving ethics training through informal networks, research supervisors, and academic workshops.	“Some competitions from big tech companies like Microsoft and Meta discussed these topics, and in our school, we also have weekly seminars about them.”
	Lack of formal training	Having little to no structured ethics education leads to uncertainty about ethical risks in AI research.	“I haven’t received much training in AI ethics, so sometimes I don’t even know what the problems are. Getting more training would help me recognize the issues and address them better.”
**Ethical encounters**			
	Data bias and fairness	Challenges related to underrepresentation in training data and unfair model outputs.	“Sometimes the data we use don’t really represent everyone, so the model ends up being unfair to certain groups.”
	Privacy concerns	Issues with using patient data without proper consent or beyond the original intended use.	“Using patient data can be tricky; we’re not always sure if we have full consent or if it’s okay to reuse it for other purposes.”
	Use of generative AI	Concerns about researchers using tools like ChatGPT to fabricate or skip steps.	“Some people just ask ChatGPT to write sections for them, and that really blurs the line between help and fabrication.”
	Commercialization pressures	Ethical concerns regarding profit-driven deployment by tech companies over academic integrity.	“Once big companies get involved, the focus often shifts from research integrity to making profits.”
	Focus on accuracy over ethics	Some researchers prioritize performance over ethical considerations.	“Everyone talks about model accuracy, but barely anyone mentions the ethical side of it.”
**Reflections on ethical implications**			
	Model generalizability and explainability	Ethical concerns arise when models cannot be applied broadly or are not easily interpretable.	“Sometimes the model works great on one dataset but fails completely on another, and we don’t really know why.”
	Impact on patient care	Researchers worry about AI models causing harm or failing to help diverse patient populations.	“If the model gives the wrong prediction, it could actually harm patients instead of helping them.”
	Clinician autonomy and displacement	Fears that AI may replace doctors or alter clinician-patient relationships.	“Some doctors worry that AI might start making decisions for them or replace parts of their job.”
	Technological pace vs evaluation speed	Difficulty in evaluating AI tools quickly enough to match their development speed.	“AI is moving so fast that our evaluation methods can’t really keep up.”
	Ethical burden on researchers	Responsibility to address AI ethics falls heavily on developers without adequate support.	“We’re the ones expected to think about ethics, but no one really gives us the tools or training to do it properly.”
**Strategies to mitigate ethical concerns (implementation and confirmation)**			
	Guideline communication	Improve access to updated ethical AI guidelines and standards.	“We really need clearer and more accessible guidelines on AI ethics; sometimes it’s hard to even find the latest ones.”
	Ethics checklists and scenarios	Using predefined lists or cases to test and evaluate model ethics and bias.	“Having a checklist or real cases to go through would make it easier to see where our model might go wrong ethically.”
	Data collaboration and diversity	Partnering with other institutions to diversify datasets and reduce bias.	“If we could share data across more institutions, the models would be less biased and more reliable.”
	IRB^a^ support and governance	Having AI-specific ethics experts within IRBs to guide responsible research.	“It would help a lot if IRBs had someone who actually understands AI to guide us on the ethical parts.”
	Inclusion of bioethicists	Adding bioethics experts to AI teams to help identify and resolve ethical issues.	“Having a bioethicist on the team would make us think about these issues more seriously from the start.”

^a^IRB: Institutional Review Board.

### Medical AI Ethics Knowledge Acquisition

Participants discussed various avenues through which they sought AI ethics information and knowledge, including peer-reviewed publications, journal feedback, social media, and informal institutional learning. Several participants mentioned journal submission guidelines or peer review feedback that relayed information on AI ethics or included statements that mentioned AI use and plagiarism:

Although I had studied ethics during my medical school, I never paid attention during machine learning research. The first time I came to know was when I submitted my manuscript…the first thing they [reviewers] pointed out was about this [ethics]…most of the reviewers were concerned about it. So that’s when I started thinking about it more seriously. I came to start thinking about ethics because if you publish in good journals, people will point out those things.

P2

Others observed a rise in publications on AI ethics and begun to read peer-reviewed articles for information: “I started seeing the trend of new publications coming up, talking about bias, fairness, and then AI ethics” (P5).

Other participants relied on social media to inform AI ethics knowledge:

I’ve been following [on X] anyone that has their hands in AI ethics or AI policy and checking all the guidelines, not just institutional levels, but national and international levels. It’s hard to keep up with all the literature that’s being pumped out right now. But it’s important to at least familiarize yourself with some of the different pieces…and what the relevant concerns are that are transcending that international sphere.

P6

Social media was perceived as more current and relevant than peer-reviewed publications, able to keep up with fast-paced developments.

A small number of participants described that AI research ethics knowledge derived from informal discussions with supervisors or participation in university seminars or workshops:

I have really lucked out into having good people in my circle and training me. I think that’s a huge resource in terms of understanding ethics and AI, and then also intentional engagement with current guidelines that are being put out.

P6

Yet, one participant remained silent during this discussion. When prompted, the participant stated having little to no knowledge about AI ethics:

I have received not too much training in AI ethics, so that is why I don’t even know what the problems are. Even if I’m making some mistakes, I don’t know if those are things that I should have been careful about.

P2

This participant’s experience is important to elucidate, as it shows potential training gaps and impacts on students and researchers.

### Ethical Encounters in AI Development

Participants reflected on ethical challenges encountered in their research environments, including concerns about data bias, privacy, commercialization, and the use of generative AI tools. Participants discussed the fabrication of data, in which the reliance on AI-generated tools, like ChatGPT, has enabled colleagues to skip steps through automated written responses. Yet, the primary ethical obstacle in medical AI research, as reported by participants, was bias and underrepresentation within training datasets. The ethical issues were consequences related to predictive modeling and fairness, especially how data omission could disproportionately exclude people of color. As a participant who worked on radiation therapy, observed:

When we build the predictive model, if our model is just purely based on the data we collected, it seems like it’s not very fair for Black people or Asian people. That’s the issue we are currently facing.

P4

Another participant agreed, stating:

Most of the data are coming from European [and] the therapeutics will be ultimately optimized for a certain group of people [so] it can’t be generalized. If you are not careful with what kind of metric you’re using to assess and evaluate that model, you’re pretty much classifying everything as negative. And institutions like to incorporate these models into their systems. If you put more weight on them [the positive cases] you might be identifying the white skin tone but not the darker skin tone.

P1

There were also concerns of data security and the risk of breaching patient data privacy:

If you’re using patient data without their proper consent or you use data that trains a model that is then used for something else that’s not within the previously defined scope, that is not ethical.

A participant discussed that privacy was also the “need to test it [the model] and then be transparent to the community and [provide] the proper instructions of the model’s performance degree” (P10) to ensure that the model is explainable and interpretable to key stakeholders.

Participants deliberated on the dangers of the commercialization of AI technologies and limited regulations:

What I am really concerned with is that these big tech companies are pushing very hard to deploy their AI model into the hospital system. It’s linked to profit, a very profitable market; if those big tech companies want to push their product, I don’t know whether they will do it with the same level of checks and balances because it’s profit-driven, and they can promise a lot of money, and we cannot make that promise.

P9

The overall ethical concern related to commercialization was the fast-paced development of AI and the time sensitivity of implementing AI into health care spaces; for-profit companies will be selected over evidence-informed AI research programs.

The perceived competitiveness between big tech corporate research and also academic research and I feel like they are not playing by the rules because they can skirt and essentially do things that we have to abide by like privacy issues and so forth.

P8

In contrast, there were participants who did not perceive these as ethics issues but rather as an accuracy issue: “I’m not really focused on ethics. I tend to focus more on accuracy, something that will make the model better but not actually the ethics” (P3).

### Reflections on Ethical Implications

Participants described how the ethical encounters stated above influenced research design and modeling choices and raised broader concerns about patient care, clinician roles, and the future of health care. For example, issues related to fairness and bias influenced generalizability:

from a data scientist perspective, it’s an issue; you cannot have a very accurate model with very high bias. You can build your model, but we want the model to have higher generalizability; we need to take this issue from a data scientist perspective.

P4

Participants noted that limits to explainability of AI impeded solutions to resolve ethical issues:

with so much advancement in AI technology, there is still no standard correct ways of evaluating my model because I haven’t understood my data or the distribution of the data yet.

P1

Other participants considered ways ethical issues in AI development may impact patient care and physician interactions. For example, participants who worked on large language models deliberated on how AI tools can generate clinical notes for the patient and questioned the accuracy of how “clinical notes could be to the specific patient…. we don’t know how that benefits the patients” (P7). Questions related to predictive modeling also drew fears of perpetuating patient harms:

How do we balance advancing healthcare to truly help patients in this unprecedented way, but also make sure that we’re not exploiting them or using models that aren’t appropriate for them?

P6

Participants reported that physician autonomy and patient-physician relationships were another important area to identify the ethical implications of AI deployment in health care. One participant asked, “whether AI is going to replace certain jobs and tasks and maybe even eventually replace doctors; that’s a conversation I have about my research” (P8). The timing to evaluate technology was an added concern that could impact patient care:

By the time you come up with a standard metric that you need to satisfy your AI model to be deployed in a healthcare facility…maybe the technology has completely changed by then. I don’t know what the solution would be, but clinicians, researchers, lawmakers, you know, everybody needs to be on board because they can no longer take that long to evaluate a technology.

P1

The perceived impacts of AI ethics placed added burdens and responsibilities on AI researchers and developers. Heightened attention on AI ethics placed more obligations on AI developers and researchers to resolve these issues, yet without training or learned mitigation strategies.

### Strategies to Mitigate Ethical Concerns

Participants described a range of strategies to address AI ethics in research, including individual practices, team-based approaches, and institutional-level interventions. Participants suggested individual and team-based approaches that foster transparent communication and knowledge mobilization. For example, a participant emphasized the need for

…good communication about the latest guidelines that are available from different communities. If that becomes available to use as students and even as faculty that will be more helpful to make us more compliant with those regulations.

P5

Guidelines, in turn, can assist in the design and development of checklists or critical scenarios to mitigate biased models. One participant stated

…maybe we can have a checklist on what we need to see before doing something that’s more concrete. I know it’s difficult to do that in AI ethics because we don’t directly use it for patient outcomes right now. But I think it will be a good starting point to have some kind of checklist on what we should be careful about.

P2

Another participant wanted the actual model to counteract biases:

The first check should be done on the data and how the data biases have been handled by the AI models. And last, what are the abusive ways this model can be used? We should have some critical scenario by which we can test our model, like some exerted test on the product to see whether this product is stable up to two years…whether it is up to our expectation.

P1

Participants advocated for organizational and institutional strategies to support ethical AI development. One approach mentioned was multi-institutional collaboration to improve data quality and diversity and mitigate bias by increasing access to larger, more representative datasets. One participant said: “Where you don’t have enough data to support the deep learning [models], we have to collaborate with other institutes to not only expand the sample size but also introduce diversity into the data” (P4). Participants also called for ethics consortiums to foster ethical awareness and skill development. For instance, a participant said: “I feel like getting more training will help me more to even identify what the problems are and then address them” (P2).

An added strategy was to equip the IRB with AI-specific guidance or AI ethics expertise on regulatory committees to enhance regulation through a uniform approach and facilitate adherence to best practices. IRB involvement could promote a more consistent approach to oversight and improve adherence to best practices. However, several participants expressed frustration that current assistance or guidance sought from the IRB often resulted in confusion rather than clarity:

We have those IRB boards and maybe some better governance…to have somebody also on AI ethics and being responsible for sharing that awareness as well as ensuring that we are going through the guidelines and sticking through the regulations.

P5

Finally, for some participants, a bioethicist or ethics expert should be involved as a potential interdisciplinary member of the research team:

When you don’t have a bioethicist at your beck and call or infused in the research in some degree, that makes it really tricky too because you might not have the checks and balances that are appropriate in maybe expanding your research or getting into the right market.

P6

## Discussion

### Overview

The integration of medical AI in preventive care and clinical decision-making means that researchers, data scientists, and those involved in the design and development of AI need to start becoming attuned to its clinical impacts. This study aims to address gaps in scholarship by examining AI researchers’ ethics perspectives. Academic institutions, such as universities and research institutes, play a central role in educating the future generation of AI developers on AI ethics and design.

Findings from the study inform how medical AI may be diffused into health care settings and how its use communicated effectively between physicians, staff, and patients. The themes from this study may be adapted into Rogers’ diffusion of innovation framework [29]. Rogers maps a 5-stage decision-making process to evaluate and adopt AI innovation into practice. The series of stages is as follows: (1) knowledge (gains understanding), (2) persuasion (reflect on attitudes), (3) decision (activities and experiences that lead to choice), (4) implementation (its actual use in practice), and (5) confirmation (to avoid dissonance and conflict).

Our findings ought to be conceptualized within the diffusion of innovation framework to understand how perspectives of AI researchers and developers offer insight into the steps, attitudes, and barriers that influence the decision-making of medical AI adoption and integration. Findings from this study may inform policy, practice, and education efforts to readily prepare AI researchers and developers to identify and examine ethical encounters in their work and to illustrate how medical AI attitudes, perceptions, and support may influence adoption or rejection [29].

### AI Ethics Knowledge Acquisition

In the knowledge stage of individual decision-making, participants in this study received information about AI ethics from a multitude of sources, including social media, peer review journal commentary and publications, and voluntary workshops and seminars. Students’ particular focus on social media as an access point for AI knowledge may be an important consideration to assess (1) the type of accuracy of messaging received and (2) the ethical issues being described and disseminated. Knowledge garnered through social media may filter into how students understand and evaluate their own research and ethical encounters, including how early adopters may rely more on social media than on peer-reviewed sources. For example, participants who described issues as rooted in accuracy rather than ethics may benefit from conversations and messaging that deciphers ethical issues from technical issues; the ability to understand how to identify and label issues as ethical (rather than solely technical) could enhance medical AI ethics knowledge and lead to a more nuanced and robust deliberation on how ethics may impact medical AI design, development, and deployment.

### Ethical Encounters and Resolution Strategies

Past research experiences of participants impacted their attitudes and perspectives of medical AI. Bias and fairness were central ethical challenges identified by participants, particularly the underrepresentation of people of color in training datasets. Such omissions risk reinforcing structural inequities and limiting the generalizability of medical AI systems. To address these issues, future research should prioritize diversifying medical datasets and integrating fairness auditing across development, supported by multi-institutional collaborations and community engagement to ensure representativeness, transparency, and accountability [30-32]. Together, these efforts can help mitigate the disproportionate exclusion of marginalized groups and promote more equitable AI-driven health outcomes to address negative attitudes toward medical AI innovation.

Extant scholarship echoes the current study’s findings by demonstrating that AI developers possess some awareness of key ethical principles, such as fairness, data security, transparency, and reliability [14], that may persuade their decision-making in adoption. Nichol et al [14], for example, conducted semistructured interviews with 40 employees from AI organizations. Participants in the study identified potential impacts of ethical issues, such as violation of patients’ privacy, misdirected health care practices, and disrupted health care systems. Other studies have similarly shown that some AI developers are sensitive to broader societal impacts of ethical AI [24,25], beyond technical issues and optimization of their algorithms [33,34].

Although participants in this study were able to identify emerging ethical issues and had thoughtfully evaluated how these issues would impact patient and clinician experiences, there were limited resolution strategies. These experiences impacted AI researchers’ decision-making, including not knowing whether to adopt or reject innovations in their work. For participants, ethical encounters were often left unresolved with no clear direction on how to proceed. The lack of informed decision-making was rooted in a lack of clarity from institutions and left participants feeling that AI researchers and developers held an undue burden in deploying ethical AI tools without further scrutiny or analysis. The added pressure on AI developers and researchers to perform was perceived as a challenge, and our findings suggest that other key stakeholders (including physicians or clinicians and health systems) ought to contribute to ethical decision-making when AI is used in practice. Thus, findings from this study show that decisions of whether to adopt or reject AI ought to include diverse perspectives to allow for more information, to identify problems, and to have support [29].

AI models must continue to be questioned and analyzed by stakeholders even after deployment. With AI technologies changing so rapidly, participants struggled to balance the fast-paced development of AI algorithms with the ethical concerns that arose. This led participants to articulate that medical AI tools ought to be continuously reviewed and evaluated.

The barriers and limited support indicate that implementation and confirmation, the final stages in the innovation-decision process, may be difficult to reach. Participants described that in the development of medical AI innovation, they often evaluated long-term impacts on patients and families and desired further support from mentors, supervisors, and organizational leadership. The perspectives of participants show that there continue to be conflicting messages and dissonance among researchers and developers regarding the adoption and use of medical AI in practice settings. Further organizational practices and policies ought to be considered to assist in decision-making activities to facilitate a more robust and comprehensive adoption process.

This study’s findings echo prior work wherein AI developers voiced confusion regarding their own roles and responsibilities in mitigating the potential harm of their tools, compounded by perceived limited authority, external pressures to produce, and the difficulty of balancing productivity and ethical considerations [26,33]. Algorithm development is highly complex and iterative, making it difficult for researchers to predict its ethical impact and apply oversight in the process. As participants in this study noted, transparency and explainability were key ethical issues, and a gap in accessible checklists or guidelines heightened obstacles to elucidating datasets and explaining patterns to clinicians and patients who may rely on these algorithms for diagnosis and treatment. The issue here is that resolving ethical encounters requires additional time and energy from AI developers, which may be an added challenge in a high-stress environment that is at odds with the fast-paced development of commercial AI tools [14]. The capacity to build collaborative environments, hold ethics consortiums, and have a robust network of people and resources to support AI ethics awareness, knowledge, and action is critical to support AI developers and researchers. Relieving some of the burdens on AI developers and researchers with institutional mechanisms can model an environment that supports ethical rigor and deliberation and lead to reinforcement and confirmation of medical AI technologies in practice settings.

As pointed out by Mittelstadt [35], compared with medicine, the field of AI research is much more heterogeneous, without defined common aims, fiduciary duties, or historical professional norms. The constant changes and shifts related to AI policy and procedure create difficulties in outlining consistent guidelines or measures to follow. Additionally, AI developers typically have backgrounds in computer science with limited training in ethics. The relative unfamiliarity with ethical principles and their implications could add further barriers to ethical medical AI development, potentially leading to ethically flawed AI products that could impose unintended harm to patients [34]. Thus, multisite collaborations, interdisciplinary communication, and IRB guidance and best standards may help to reduce the burden on AI developers, create more teachable moments, and establish more thoughtful and intentional mechanisms for deliberating ethical encounters, along with clear resolution pathways to facilitate implementation and confirmation.

### Bioethics-Informed Guidance

The inclusion of bioethicists on research teams, as stated by participants, has been suggested in prior theoretical scholarship. For example, McLennan et al [36] proposed the concept of “embedded ethics,” a collaborative approach that creates interdisciplinary research teams whereby AI developers and ethicists can anticipate, identify, and address ethical issues as they arise in the development process. Other studies have suggested a practical ethics checklist for AI developers [37] that recognizes ethical and social responsibility within AI development [38] or ethics guidelines and review processes specifically designed for AI developers [39,40] to support research design and analysis. These efforts offer improvements by having a refined focus on the practicality of how to use ethics recommendations and an emphasis on frontline AI developers, who can help mitigate ethical issues prior to AI deployment and use. This study’s findings demonstrate that AI developers are interested in gaining knowledge about AI ethics, are already deliberating on the ethical encounters in their design and development, and are thoughtful about the longer-term practical implications of their work in health systems. Future research ought to consider strategies to mitigate ethical encounters and to advocate for heightened ethics knowledge, training, and conversations specifically targeted for AI developers and researchers. Foundational seminars on how to identify and label an issue as ethical (as opposed to technical) are a critical first step in training to ensure all developers and researchers can recognize these encounters in practice. Supervisors and managers must consider ways to encourage ethical dialogue and empower students and faculty to seek ways to mitigate ethical concerns and bridge their work to practice. This may involve bringing in bioethicists or other ethics experts who can speak diligently, thoughtfully, and comprehensively about these topics. AI developers and researchers should not be working in siloes but rather placed in communities with other medical AI stakeholders to heighten ethical dialogue and theorize novel mitigation strategies. Additionally, to enhance the actionability of these recommendations, institutions could develop sample ethics checklists (eg, addressing data representativeness, model explainability, and patient privacy), workflow templates that map ethical review points within the AI development process, and metrics to evaluate ongoing compliance. Such practical tools can help translate ethical principles into consistent, operational practices for research offices and AI teams. These steps may facilitate the diffusion of innovation processes to allow for an easier and more transparent decision, implementation, and confirmation process that can lead to ethical adoption of medical AI. It is clear that ethical conflict and encounters of ethical dilemmas in the development and deployment of medical AI have stark impacts on the diffusion of innovation and the ability to effectively implement and reinforce the decision to adopt. Future research may seek to understand how this process may infiltrate the decision-making and ethical attitudes and perspectives of other key stakeholders, including physicians, allied health workers, health care administrators, patients, and families.

### Limitations

This is one of the first studies in North America to examine AI developers’ knowledge, encounters, and recommendations of medical AI ethics. Yet, there are several limitations. The small and relatively homogenous sample limits the diversity and generalizability of the findings. Future research should pursue broader and more inclusive investigations that capture perspectives from a wider range of disciplines, institutions, and demographic backgrounds across the United States. The representation of only 5 academic institutions may narrow the findings and may overlook other ethical concerns that emerge in distinct research areas. This was also a qualitative focus group study, and, thus, participants may have had concerns regarding privacy and confidentiality, reputation, and status in responses, and the emergence of potential power imbalances with student participants. Furthermore, as with most focus group studies, participants may have provided more socially desirable responses due to the group setting or the presence of peers, which could have influenced the depth or candor of some discussions. The focus group facilitator mitigated any ethical concerns by setting group norms, ensuring privacy and confidentiality, and piloting focus group questions and prompts. Future research may consider an anonymous survey to broadly examine ethical encounters in medical AI research.

### Conclusions

As an exploratory pilot study, the current findings provide preliminary insights that can guide future empirical and institutional efforts. Findings from this study are important to determine the next steps to facilitate ethical decision-making among medical AI developers and researchers. There ought to be strategies to effectively deliberate about AI ethics across research teams and create opportunities for multisite collaboration, IRB debriefs and guidelines, protocol checklists and testing mechanisms, and the involvement of key stakeholders in deliberation, including bioethicists, clinicians, patients, and hospital leadership or administration with AI research teams. These initial insights lay the groundwork for larger-scale, multi-institutional investigations that can further validate and expand on the patterns identified here. The perspectives of key stakeholders may inform stages in the innovation-decision process and gain insight into barriers, supports, and resources necessary to ethically adopt medical AI into practice.

## Appendix

Multimedia Appendix 1 COREQ Checklist.

## Abbreviations

AI: artificial intelligence
EHR: electronic health record
IRB: institutional review board

## Data Availability

The data that support the findings of this study are not publicly available due to privacy and confidentiality agreements.
